# Age, Sex, and Socio-Economic Status Affect the Incidence of Pediatric Spinal Cord Injury: An Eleven-Year National Cohort Study

**DOI:** 10.1371/journal.pone.0039264

**Published:** 2012-06-22

**Authors:** Li-Chien Chien, Jau-Ching Wu, Yu-Chun Chen, Laura Liu, Wen-Cheng Huang, Tzeng-Ji Chen, Peck-Foong Thien, Su-Shun Lo, Henrich Cheng

**Affiliations:** 1 Department of Surgery, National Yang-Ming University Hospital, I-Lan, Taiwan; 2 Department of Surgery, School of Medicine, National Yang-Ming University, Taipei, Taiwan; 3 Institute of Pharmacology, National Yang-Ming University, Taipei, Taiwan; 4 Department of Neurosurgery, Neurological Institute, Taipei Veterans General Hospital, Taipei, Taiwan; 5 School of Medicine, National Yang-Ming University, Taipei, Taiwan; 6 Department of Medical Research and Education, National Yang-Ming University Hospital, I-Lan, Taiwan; 7 Institute of Hospital and Health Care Administration, National Yang-Ming University School of Medicine, Taipei, Taiwan; 8 Department of Ophthalmology, Chang Gung Memorial Hospital, Taoyuan, Taiwan; 9 College of Medicine, Chang Gung University, Taoyuan, Taiwan; 10 Department of Pediatrics, National Yang-Ming University Hospital, I-Lan, Taiwan; Chancellor College, University of Malawi, Malawi

## Abstract

**Background:**

Few studies focus on pediatric spinal cord injury (SCI) and there is little information regarding the cause, anatomic level, and high risk population of SCI in children. This study aims to investigate the incidence and risk factors of pediatric SCI.

**Methods:**

A nationwide cohort of 8.7 million children aged<18 years in an 11-year period was analyzed for causes, age at injury, anatomic sites, disability, and familial socio-economic factors. Incidence rates and Cox regression analysis were conducted.

**Results:**

A total of 4949 SCI patients were analyzed. The incidence rates of cervical, thoracic, lumbar, and other SCI were 4.06, 0.34, 0.75, and 0.85 per 100,000 person-years, respectively. The proportional composition of gender, age, and socio-economic status of SCI patients were significantly different than those of non-SCI patients (all *p*<0.001). Male children were significantly more likely to have SCI than females in both the cervical and the other SCI groups [Incidence rate ratio (IRR) = 2.03 and 1.52; both *p*<0.001]. Young adults and teenagers were also significantly more likely to have SCI than pre-school age children in the cervical SCI (IRR = 28.55 and 10.50, both *p*<0.001) and other SCI groups (IRR = 18.8 and 7.47, both *p*<0.001). Children in families of lower socio-economic status were also significantly more likely to have SCI (*p*<0.05).

**Conclusions:**

In the pediatric population, the overall SCI incidence rate is 5.99 per 100,000 person-years, with traumatic cervical SCI accounting for the majority. The incidence rate increases abruptly in male teenagers. Gender, age, and socio-economic status are independent risk factors that should be considered.

## Introduction

Spinal cord injury (SCI) is a catastrophic medical condition requiring chronic care. Life expectancy after SCI is reduced due to complications like pneumonia, septicemia, urinary and cardiac diseases, and proportional to the severity of injury or remaining neurologic function. [1], [2], [3], [4] For those injured as children, the life expectancy may be lower, 50–83% of normal. [5] Thus, given the early occurrence, pediatric SCI patients substantially require even more long-term care than adults.

The exact incidence rate of pediatric SCI is unclear. Reported data quotes 1–10% of all spinal injuries happen in children, with a variety of estimated incidences ranging from 1.9 to 19.9 per million children. [6], [7], [8], [9] Effective prevention of injuries in children is a huge save in healthy person-years and socio-economic cost. However, due to the scarcity of SCI in children and the lack of population-based database for investigation, the causes, patterns of injury, and risk factors remain speculative. Moreover, different vulnerabilities and patterns are inferred for pediatric SCI compared to adults due to incomplete ossification, ligamentous laxity, and distinct co-morbidities. For example, spinal cord injury without radiographic abnormality (SCIWORA) is a unique pattern of SCI described exclusively in children, depicting patients with signs of myelopathy but without radiographic evidence of spinal column injury on plain radiograph or computed tomography. [10], [11], [12], [13] As such, specific data of pediatric SCI patients in terms of anatomic site of injury, proportional distribution of age and gender, causes, and socio-economic status are invaluable for future care and prevention.

This study aimed to describe patterns and risks of SCI in children and young adults using a nationwide pediatric cohort in a developed economy (i.e. Taiwan). Its comprehensive coverage of the health care system and continuous observation for more than a decade provide a unique opportunity to examine specific cases of injury, as in a natural experiment, allowing study of individual risk factor and health economics. To date, this is the largest cohort of pediatric SCI. The data presented in the present study can be of substantial value in both medical care and health policy.

## Methods

This study used the National Health Insurance Research Database (NHIRD), a database of all claims data from Taiwan’s National Health Insurance (NHI) provided by the National Health Research Institutes (NHRI) of Taiwan. This government-run monopolistic health insurance provided universal coverage to over 26 million population since January 1996. It offered unrestricted access to any healthcare provider of the patients’ choice and thus, would reflect the reality of disease incidences and medical service utilizations.

Because individual and hospital identifiers were unique to the research database and researchers, and could not be used to trace individual patients or health service providers, this study was exempt from full review by the Institutional Review Board of National Yang-Ming University Hospital (No. 2010A024). Moreover, the Bureau of NHI of Taiwan performed a cross-check and validation process of the medical charts and claims, which ensured the fidelity of the NHIRD.

### Study Population

In the entire 11 years from January 1, 1998 to December 31, 2008, all in-patient data from NHIRD were collected for analysis. This population-based open cohort included Taiwan’s entire 0–18 years age population of 8,747,434 persons.

### Identification of SCI

Diagnoses were recorded in the NHIRD according to the International Classification of Disease, 9th Version (ICD-9). Incidences of SCI were identified as subjects who were newly-hospitalized with discharge codes of 952.x (i.e. spinal cord injury without evidence of spinal bone injury) or 806.x (i.e. fracture of vertebral column with spinal cord injury) during the study period. Subjects with any prior SCI-related admission in 1997 were excluded. The date of admission was designated as the date of SCI incidence, from which the age of SCI was derived. The incidence rates in the study were estimated by incidence density.

Subjects were censored only at the event of SCI or the end of study. All SCI events were grouped according to the anatomic level of injury using the ICD-9 codes. There were four groups of anatomic sites of SCI, including cervical (952.0, 806.0–1), thoracic (952.1, 806.2–3), lumbar (952.2, 806.4–5), or other levels including multiple sites (952.x, except 952.0–2, and 806.x, except 806.0–5, or multiple levels). Due to the scarcity of cases, the latter three were combined for analysis. Thus, there were the cervical SCI group and the other SCI (i.e. thoracic, lumbar, and others) group in the risk analysis.

### Identification of Causes of SCI

Pediatric SCI resulted from traumatic causes were grouped according to the external codes of ICD-9 of their hospitalization records. For subjects who were hospitalized with definite external causes, the causes should be explicitly recorded in up to two ICD-9 external codes in addition to diagnosis codes. Through the regular reviewing process, the correctness of such external codes of causes of traumatic incidences was examined and health providers would be penalized if the causes coded accurately. In this study, SCI hospitalization with definite external codes, defined as traumatic SCI, were analyzed and the cause of traumatic SCI were categorized into four groups including vehicle accidents (E810–E848), falls (E880–E888), other accidents (E890–E928), suicides (E950–E969) and others (the rest of E-codes).

### Demographics and Covariates

Gender, three age groups (i.e. pre-school age, school age, and teenagers), and other socio-economic characteristics (e.g. household income levels, geographic location of residency and urbanization level of households registration) were analyzed and compared to a comparison cohort composed of individuals without SCI.

Household income levels were grouped into four categories of insurance premiums: ≥NTD$40,000, 20,000–39,999, 1–19,999, and dependents (in the NHI of Taiwan, premiums were mostly income-related, which could be taken as proxy for income). Those without salaries like the unemployed, students, or the elderly were designated as dependents by the BNHI and the government. Geographic location of residency was classified into four regions as northern, central, southern, and eastern Taiwan, as in previous NHIRD studies. [14], [15] The northern region has more cities of economic and political importance, whereas the eastern region has less.

For the degree of urbanization, the location of NHI registration was used as another proxy parameter for socio-economic status. Similar to previous reports, urbanization levels in Taiwan were divided into 7 strata in which level 1 referred to as the “most urbanized” and level 7 as the “least urbanized”. However, given that there were very few SCI incidences in levels 5, 6 and 7, these three were combined into a single group and thereafter referred to as level 5. [16] Common co-morbidities (e.g. skull fractures, spinal disorders, brain cancer, and lymphoma) were adjusted.

### Statistical Analysis

All of the data were linked using the SQL server 2008 (Microsoft Corp.) and analyzed by the SPSS software (SPSS, Inc., Chicago, IL). Chi-square goodness-of-fit test equality among groups and Cox multivariate regression model were employed. A two-tailed level of 0.05 was considered statistically significant.

## Results

**Figure 1:**
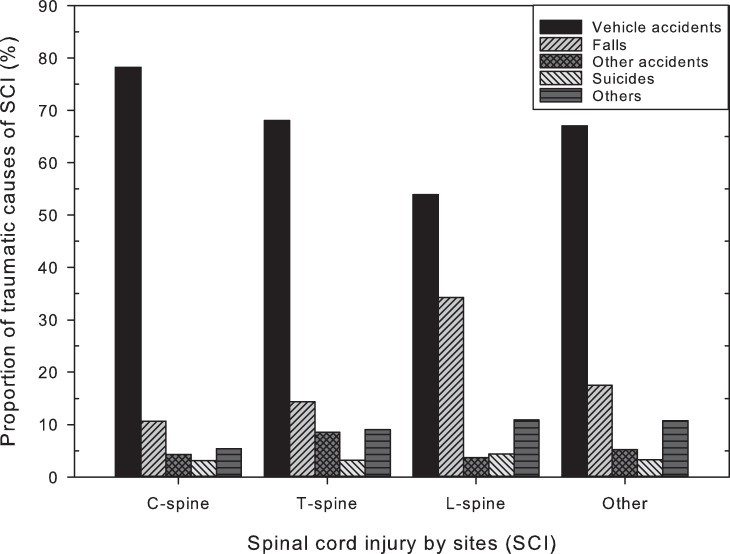
Proportional composition of traumatic causes of spinal cord injury. C, cervical; T, thoracic; L, lumbar.

From the cohort, 4949 SCI patients were identified in 82,616,952.0 person-years. There were 3351, 277, 616, and 705 patients had cervical, thoracic, lumbar, and other SCI, respectively. The comparison cohort (i.e. no SCI) was composed of 8,742,485 individuals (Fig. 1). The overall incidence rate was 5.99 per 100,000 person-years (95% CI. 3.92–4.19). The injury-site-specific incidence rates were 4.06, 0.34, 0.75, and 0.85 per 100,000 person-years for cervical, thoracic, lumbar, and other SCI, respectively (Table 1).

**Table 1:** Incidences of spinal cord injury (1998–2008).

Events	Site	Incidence Rate†	95% CI
3351	Cervical	4.06	(3.92–4.19)
277	Thoracic	0.34	(0.30–0.38)
616	Lumbar	0.75	(0.69–0.81)
705	Others	0.85	(0.79–0.92)
4949	Total	5.99	(5.82–6.16)

† per 100,000 person-year.

### Characteristics of SCI

Among the 3351 (67.7%) cervical SCI patients, there was a significant male predominance than in the comparison cohort (68.5% vs. 52.0%, *p*<0.001). The proportional composition of age in the cervical SCI group was significantly different than in the comparison group (*p*<0.001). Teenagers accounted for 69.6% of the cervical SCI group but only 25.5% in the comparison group. Only 5.0% were of pre-school age in the cervical SCI group but 48.7% in the comparison group. The proportional composition of insurance levels, geographic locations, and urbanization levels were significantly different between the two groups (*p*<0.001, *p*<0.001, and *p*<0.001, respectively).

The 1598 (32.3%) other SCI patients (i.e. thoracic and, lumbar, and others) had similar patterns as the cervical SCI group when compared to non-SCI (comparison) group. The other SCI group had more male predominance than the comparison (61.9% vs. 52.0%, *p*<0.001) and more teenagers (71.7% vs. 25.5%, *p*<0.001). Insurance level, geographic location, and urbanization levels of the other SCI patients were also significantly different (*p*<0.001, *p*<0.001, and *p*<0.001, respectively) (Table 2).

**Table 2:** Characteristics of the spinal cord injury group versus the comparison cohort.

	Comparison		Cervical SCI			Other SCI		
	n = 8742485	(%)	n = 3351	(%)	*p* value	n = 1598	(%)	*p* value
Gender					<0.001			<0.001
Female	4196229	(48.0)	1056	(31.5)		609	(38.1)	
Male	4546256	(52.0)	2295	(68.5)		989	(61.9)	
Age at enrollment					<0.001			<0.001
Pre-school age	4256368	(48.7)	166	(5.0)		88	(5.5)	
School age	2253179	(25.8)	854	(25.5)		364	(22.8)	
Teenagers	2232938	(25.5)	2331	(69.6)		1146	(71.7)	
Socio-economic status								
Insurance level (NTD$)					<0.001			<0.001
40,000-	309151	(3.5)	236	(7.0)		117	(7.3)	
20,000–39,999	2217036	(25.4)	1413	(42.2)		694	(43.4)	
1–19,999	4614416	(52.8)	1330	(39.7)		634	(39.7)	
Dependent	1601882	(18.3)	372	(11.1)		153	(9.6)	
Geographic location					<0.001			<0.001
Northern	4299585	(49.2)	1368	(40.8)		611	(38.2)	
Central	1710235	(19.6)	795	(23.7)		380	(23.8)	
Southern	2531266	(29.0)	1062	(31.7)		559	(35.0)	
Eastern	201399	(2.3)	126	(3.8)		48	(3.0)	
Urbanization level					<0.001			<0.001
1 most urbanization	2522776	(28.9)	686	(20.5)		330	(20.7)	
2 more	2724836	(31.2)	1049	(31.3)		475	(29.7)	
3 moderate	1482440	(17.0)	595	(17.8)		248	(15.5)	
4 less	1217024	(13.9)	606	(18.1)		309	(19.3)	
5 least urbanization	795409	(9.1)	415	(12.4)		236	(14.8)	

Pre-school age: 0–5 years; School age: 6–12 years; Teenagers: 13–18 years.

### Causes and Disabilities of SCI

A total of 3705 (74.9%) of SCI had known traumatic causes, including vehicular accidents, falls, other accidents, and suicide attempts. Vehicular accidents were unanimously the most frequent cause of traumatic SCI in every anatomic site of injury (79.7%, 68.1%, 53.9%, and 67.0% for cervical, thoracic, lumbar, and other SCI, respectively). The proportion of specific traumatic causes in each SCI site was shown in Figure 1.

There were 3790 (76.6%) SCI patients without evidence of spinal bone injury (i.e. SCIWORA), 1110 (22.4%) with SCI and fractured vertebral column, and 49 (1%) with both. Permanently moderate and severe disabilities (e.g. paraplegia, tetraplegia, and incontinence) were noted in 7%, 47.9%, 12.0%, and 9.6% of cervical, thoracic, lumbar, and other SCI, respectively.

### Incidence Rates of SCI

**Figure 2:**
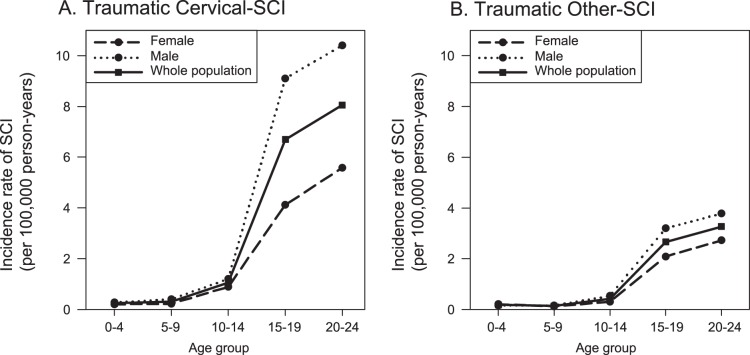
Plotted age-gender-injury site-specific incidences of spinal cord injury with definite traumatic causes. (**A**) Traumatic cervical spinal cord injury (SCI); (**B**) Traumatic other (i.e. thoracic, lumbar, and others) SCI.

The specific incidence rates of SCI with definite traumatic causes regarding anatomic site of injury (cervical/others), age, and gender were shown in Figure 2.

For traumatic cervical SCI, the incidence rates increased abruptly in teenagers. This increase was more prominent in males than in females (Fig. 2A). There were similar trends in patients with traumatic causes of other SCI (i.e. thoracic, lumbar, and other) SCI but to a lesser degree (Fig. 2B).

### Risk Analysis

After adjustments, gender, age, and several other characteristics were independent risk factors of SCI for both the cervical and the other SCI groups. Male children were significantly more likely to have SCI than females in both groups [Incidence rate ratio (IRR)  = 2.03 and 1.52, *p*<0.001 and <0.001].

Young adults and teenagers were significantly more likely to have SCI than pre-school age children in both the cervical SCI group (IRR = 28.55 and 10.50, *p*<0.001 and <0.001) and the other SCI group (IRR = 18.8 and 7.47, *p*<0.001 and <0.001).

Patients of lower insurance level, living in areas other than northern Taiwan, and less urbanization areas were significantly more likely to suffer from SCI, including both cervical and the other groups. (Table 3).

**Table 3:** Adjusted incidence ratios in pediatric spinal cord injury (n = 4949).

	Cervical SCI				Other SCI				
	IRR	(95% CI)		*p* value	IRR	(95% CI)			*p* value
Gender									
Male vs. Female	2.03	(1.89–2.19)		<0.001	1.52	(1.37–1.68)			<0.001
Age at injury									
Preschool age	*Reference*				*Reference*				
School age	1.28	(0.94–1.75)		0.118	0.91	(0.61–1.36)			0.653
Teenagers	10.50	(8.07–13.67)		<0.001	7.47	(5.42–10.29)			<0.001
Young adults	28.55	(22.04–36.98)		<0.001	18.8	(13.75–25.69)			<0.001
Socio-economic status									
Insurance level (NTD$)									
40,000-	*Reference*				*Reference*				
20,000–39,999	0.92	(0.82–1.04)		0.185	1.03	(0.86–1.23)			0.754
1–19,999	2.51	(2.24–2.82)		<0.001	2.95	(2.47–3.52)			<0.001
Dependent	1.18	(1.01–1.40)		0.043	1.46	(1.15–1.86)			0.002
Geographic location									
Northern area	*Reference*				*Reference*				
Central area	1.15	(1.05–1.26)		0.004	1.19	(1.04–1.37)			0.012
Southern area	1.09	(1.00–1.19)		0.043	1.25	(1.11–1.41)			<0.001
Eastern area	1.32	(1.09–1.59)		0.005	1.03	(0.76–1.40)			0.835
Urbanization level									
1 most urbanization	*Reference*				*Reference*				
2 more	1.36	(1.23–1.50)		<0.001	1.25	(1.08–1.44)			0.002
3 moderate	1.35	(1.21–1.51)		<0.001	1.13	(0.96–1.34)			0.151
4 less	1.70	(1.51–1.91)		<0.001	1.76	(1.49–2.08)			<0.001
5 least urbanization	1.78	(1.56–2.03)		<0.001	2.03	(1.69–2.44)			<0.001

Pre-school age: 0–5 years; School age: 6–12 years; Teenagers: 13–18 years; Young adults: 19 years and above.

## Discussion

This study used a national pediatric cohort composed of more than 8.7 million children to analyze SCI covering 11 years. Of the 4,949 SCI patients identified, with an overall incidence rate at 5.99 per 100,000 person-years, more than two-thirds (67.7%) were cervical SCI and a majority (74.9%) had known traumatic causes. Age, gender, and family socio-economic status were independent risk factors of SCI. Older males from lower insurance premium families who lived in more rural areas had higher risks. There was an abrupt increase in the incidences of traumatic cervical SCI in male teenagers (Fig. 2A). This report is the first comprehensive investigation of pediatric SCI on a nationwide scale, with a longitudinal span longer than a decade.

SCI is considered less frequent in children than in adults. Apple et al. report that only 5% of 1770 traumatic SCI patients are under the age of 15 years. [17] Di Martino et al. estimate that pediatric SCI and vertebral column injury represents up to 10% of all spinal injuries reported in the general population. [18] The incidence of SCI under the age of 15 years is reportedly 4.6 per million children per years in Sweden from 1985 to 1996 through 92 injured children. [19] The incidence rate is 1.99 per 100,000 children in the United States from 1997 to 2000 from a database containing 2.5 million discharge notes. [7] A study from Finland containing 749 spinal injury children from 1.1 million report a mean annual incidence of spinal injuries at 66 per million and an annual SCI incidence of 1.9 per million. [6] The substantial inconsistency of reported incidences reflects the difficulty of investigating such a rare disease. Institution-based studies with cross-sectional observation inherently lack the complete epidemiologic profiles of pediatric SCI.

The true incidence rate of pediatric SCI, especially of traumatic causes, is likely related to the community or country under investigation. Regional differences of chances of injury certainly exist among SCI caused by vehicular accidents, sports activities, or crime related events. This is a multi-factorial issue that varies tremendously over time. Accurate region- and ethnic-specific incidence rates in particular populations may improve the management and administration of healthcare policy for SCI, while understanding risk factors associated with pediatric SCI, especially modifiable ones, may be more critical for prevention.

The distribution patterns of SCI in the young have a number of features. The current study, with its longer cohort and larger number, corroborates previous inferences. Younger patients account for fewer SCI. [20], [21], [22] Although pre-school age and school age children have similar incidences of SCI in the present study, the risk ratio of SCI in this cohort increases astonishingly, 10.5 times for cervical and 7.5 times for other SCI, among teenagers. The risk continues to increase in young adults, 28.6 times for cervical and 18.8 times for other SCI, compared to pre-school age children. In addition, the incidences of both male cervical and non-cervical SCI exceed those of females after the age of 15 years (Fig. 2A and 2B). Given that male adolescents and young adults tend to participate more in many activities that put them at risk of injury, results of the present study can be reasonably inferred.

The cervical spine accounts for a majority of pediatric spinal trauma. [23], [24], [25] It is comparable that in this cohort, more than two-thirds of SCI events are cervical. The incidence rates of cervical, thoracic, lumbar, and other SCI are 4.06, 0.34, 0.75, and 0.85, respectively. The pediatric population has disproportionately higher numbers of cervical SCI than SCI of other sites. It is noteworthy that this huge discrepancy in proportional composition of cervical SCI is not demonstrable in adult SCI. [16] Several anatomic and biomechanical factors may explain the differences in injury profiles. For example, different vertebral configuration, incomplete ossification, relatively larger head size, and more ligamentous laxity can make children prone to cervical SCI. [25], [26], [27], [28]

Vehicular accidents are the most common cause. In the current cohort, vehicular accidents are unanimously the most frequent cause of traumatic SCI in all anatomic sites of injury (79.7%, 68.1%, 53.9%, and 67.0% for cervical, thoracic, lumbar, and other SCI, respectively). This is comparable to other reports of etiology. [7], [9], [29], [30], [31] Traffic accident related information (e.g. seat-belt, driver age, and law enforcement) are not accessible in the current study. Although this correlation may be quite helpful in legislation of health policy, the current database provides insufficient data. This is a limitation of the study. The second most commonly reported causes of SCI in literature are falls or sports-related injuries, and similar results are demonstrated in Figure 1. Furthermore, male children have higher risks than female. Children from families of lower socio-economic status are more likely to become victims of SCI. The fact that younger adults of lower socio-economic status living in the rural area are at remarkably higher risk of SCI, estimated to be 127.4 (95% CI = 93.4–173.8) times compared to reference, warrants attention. This information is not only important in the care of these SCI patients but also critical for health policy in the future.

The major limitation of the study is its lack of neurologic evaluations. There are no standardized records of neurologic status immediately post-injury available for analysis in the database used. The final stable neurologic status of each patient is only accessible through individual medical records. The registry of catastrophic illness patients (HV1997–2008) of the NHIRD is used as proxy for identifying patients with SCI causing moderate and severe disabilities. Therefore, the percentage of patients with severe permanent neurologic deficits like paraplegia, tetraplegia, and incontinence are all computed by linked analysis. However, the correlation of treatment and neurologic recovery in this young population is unclear. Future studies through more detailed records of neurologic functions are warranted to understand the recovery process in pediatric SCI.
